# Small cell lung cancer transformation and tumor heterogeneity after sequential targeted therapy and immunotherapy in EGFR-mutant non-small cell lung cancer: A case report

**DOI:** 10.3389/fonc.2022.1029282

**Published:** 2022-12-07

**Authors:** Meng-Hang Yang, Jia Yu, Chen-Lei Cai, Wei Li

**Affiliations:** Department of Oncology, Shanghai Pulmonary Hospital, School of Medicine, Tongji University, Shanghai, China

**Keywords:** histologic transformation, tumor heterogeneity, immunotherapy, targeted therapy, case report

## Abstract

**Background:**

Histological transformation from non-small cell lung cancer (NSCLC) to small cell lung cancer (SCLC) is one of mechanisms of the acquired resistance to epidermal growth factor receptor (EGFR)-tyrosine kinase inhibitors (TKI). However, SCLC transformation and tumor heterogeneity have never been reported in sequential targeted therapy and immunotherapy.

**Case presentation:**

Here, we described a patient with advanced EGFR-mutant NSCLC, who received erlotinib and underwent the resistance with EGFR T790M (–). The patient then received chemotherapy plus immunotherapy of programmed cell death 1 (PD-1) inhibitor, encountered progression with pathological transformation from NSCLC to SCLC that was overcome by chemotherapy of etoposide plus carboplatin (EC) with the main lesion significantly shrinking while metastatic nodules increasing. The pathology of the metastatic nodule showed NSCLC with EGFR T790M (+). Based on the tumor heterogeneity, EC chemotherapy combined with osimertinib was used, and patients responded well. The patient experienced four lung biopsies in all, which helped to provide the patient with precise treatment.

**Conclusions:**

This case suggested that SCLC transformation and tumor heterogeneity should be paid attention to when disease progression occurred in advanced NSCLC whether receiving targeted therapy or immunotherapy.

## Background

Lung cancer is one of malignant tumors with highest incidence and mortality rate. Non-small cell lung cancer (NSCLC) accounts for about 80~85% of all lung cancer. In NSCLC with epidermal growth factor receptor (EGFR) mutations treated with EGFR-tyrosine kinase inhibitor (TKI), there is a rare phenomenon of drug resistance, that is pathological type transformation. The transformation to small cell lung cancer (SCLC) is one of the important mechanisms of resistance to EGFR-TKI. In fact, it is found that SCLC transformed from NSCLC has similar clinical characteristics with primary SCLC. For patients with SCLC transformation, chemotherapy is short-term effective, and the prognosis is poor with the median overall survival (OS) less than 1 year (1, 2).

With the increase of re-biopsy in clinical practice, SCLC transformation is found to be not limited to specific molecular subtypes of NSCLC, nor to specific treatment. Immunotherapy is increasingly used in advanced lung cancer. However, the transformation of NSCLC to SCLC during immunotherapy has only been reported in a few cases (3–5). Here, we described a rare case of an advanced NSCLC patient with EGFR exon 19 deletion (19del) who has received sequential targeted therapy and immunotherapy of programmed cell death 1 (PD-1) inhibitor, and undergone a pathological transformation from NSCLC to SCLC and inconsistent gene status that revealed tumor heterogeneity. We presented this case in accordance with the CARE guideline (6).

## Case presentation

**Figure 1:**
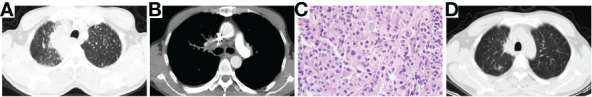
Chest CT scan of baseline **(A, B)**, pathological examination (H&E stain) showing lung adenocarcinoma **(C)** and chest CT scan of best response PR after the treatment of Erlotinib **(D)**. CT, computed tomography; PR, partial response.

A 50-year-old man was admitted to our hospital on Feb 19th, 2019, complaining of recurrent dry cough for 3 weeks. He had a smoking history of half pack per day for 20 years. Physical examination discovered that one of the left supraclavicular lymph nodes was enlarged. Chest computed tomography (CT) scan showed a mass in the upper lobe of the right lung accompanied by multiple small nodules in both lungs, enlarged mediastinal and hilum lymph nodes (**Figure 1A, B**). Enhanced brain magnetic resonance imaging (MRI) showed a lesion in the cerebellum that tended to be tumor metastasis. Bone scan and abdomen B-ultrasound showed no active findings. We performed biopsies of both the enlarged left supraclavicular lymph node and the right upper lobe (RUL) lesion. The pathology results indicated lung adenocarcinoma (**Figure 1C**), and the genetic testing found EGFR 19del mutation. The patient was diagnosed with right upper lobe lung adenocarcinoma T4N3M1b-Stage IVA (brain, contralateral lung, pleura) with EGFR 19del. Erlotinib as the first-line therapy was given, and the best response was partial response (PR) (**Figure 1D**). The progression-free survival (PFS) of first-line therapy was 6.5 months.

**Figure 2:**
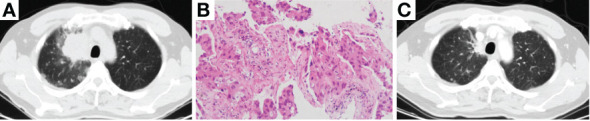
Chest CT scan of PD resistant to erlotinib **(A)**, photomicrograph of re-biopsy (H&E stain) showing lung adenocarcinoma **(B)** and chest CT scan of best response PR after the treatment of chemotherapy combined with PD-1 inhibitor **(C)**. CT, computed tomography; PD, progressive disease; PR, partial response; PD-1, programmed cell death 1.

Then the patient underwent progressive disease (PD) (**Figure 2A**). A repeat biopsy of RUL lesion revealed lung adenocarcinoma (**Figure 2B**) with EGFR 19del, T790M (–) and programmed death ligand 1 (PD-L1) tumor proportion score (TPS) 90% (+). The patient participated in a phase III clinical study (JS001-CT25-III-NSCLC), and received 6 cycles of PD-1 inhibitor (toripalimab) plus pemetrexed and carboplatin, followed by 2 cycles of toripalimab plus pemetrexed as maintenance treatment. PR was achieved again (**Figure 2C**). The PFS of second-line therapy was 6.6 months.

**Figure 3:**
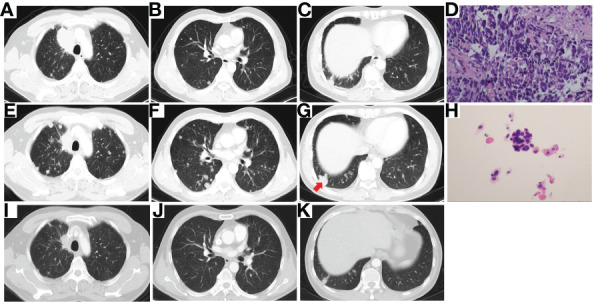
Chest CT scan of PD after 8 cycles of chemotherapy combined with PD-1 inhibitor **(A–C)**, photomicrograph of the third biopsy (H&E stain) showing SCLC transformation **(D)**, Chest CT scan after 2 cycles of EC chemotherapy showing the main lesion of RUL significantly shrank **(E)**, while intrapulmonary metastatic nodules increased and enlarged **(F, G)**, the pathology (H&E stain) of the enlarged metastatic lesion of RLL (*red arrow*) showing NSCLC **(H)**, and chest CT scan of PR after 2 cycles of combination therapy of osimertinib and EC chemotherapy **(I–K)**. CT, computed tomography; PD, progressive disease; PD-1, programmed cell death 1; SCLC, small cell lung cancer; EC, etoposide plus carboplatin; RUL, right upper lobe; RLL, right lower lobe; NSCLC, non-small cell lung cancer; PR, partial response.

Then the patient experienced the second PD (**Figures 3A–C**), and the third biopsy of RUL lesion was performed. Unexpectedly, histologic analysis showed a transformation to SCLC (**Figure 3D**) with immunohistochemical staining confirmed as Syn (+) and Ki-67 (70%+). Then, the patient received the treatment of etoposide plus carboplatin (EC). After 2 cycles of EC chemotherapy (PFS 1.9 months), the main lesion located in RUL significantly shrank (**Figure 3E**), however, the intrapulmonary metastatic nodules increased and enlarged (**Figures 3E–G**).

**Figure 4:**
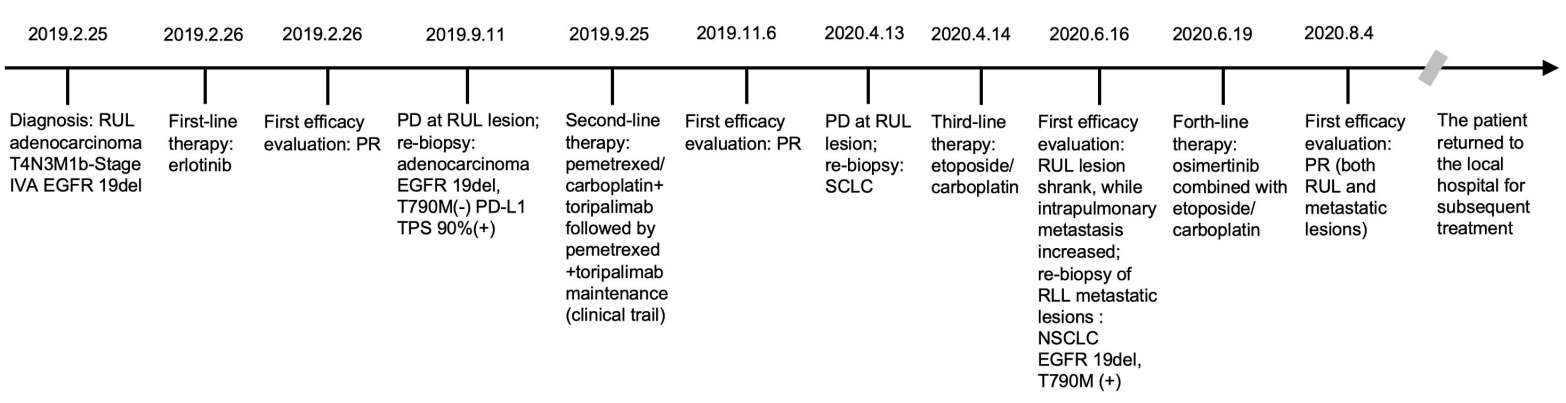
Timeline of the patient. RUL, right upper lobe; EGFR, epidermal growth factor receptor; PR, partial response; PD, progressive disease; PD-L1, programmed death ligand 1; TPS, tumor proportion score; SCLC, small cell lung cancer; RLL, right lower lobe; NSCLC, non-small cell lung cancer.

To find out the reasons for the inconsistent response of different lesions to EC chemotherapy, we performed a needle aspiration of an enlarged metastatic lesion in the right lower lobe (RLL). The pathological results revealed NSCLC (**Figure 3H**) with the genetic testing showing EGFR 19del and T790M (+). Considering the heterogeneity of tumor, after comprehensive discussion of multi-disciplinary team (MDT), the patient began to receive EC chemotherapy combined with the 3rd-generation of EGFR-TKI, osimertinib. Encouragingly, after 2 cycles of combination therapy, the main lesion in RUL continued to shrink (**Figure 3I**), and the metastatic nodules in both lungs decreased significantly (**Figure 3J,K**). PR was achieved again. The patient well tolerated with the treatment. He then returned to the local hospital for subsequent treatment. The timeline of the treatment process is shown in **Figure 4**.

## Discussion and conclusions

SCLC transformation has been recognized as one of mechanisms of resistance to EGFR-TKI in advanced lung adenocarcinoma with EGFR mutant, accounting for 5%~15% of the causes of EGFR-TKI resistance (7, 8). In contrast, transformation of NSCLC to SCLC was less reported in patients receiving immunotherapy like PD-1 inhibitors. Imakita et al. (3) reported SCLC transformation during immunotherapy with nivolumab. Abdallah et al. (4) reported two cases of potential histologic transformation of NSCLC to SCLC during the treatment of nivolumab and pembrolizumab, respectively. Iams et al. (5) described two cases of SCLC transformation as a mechanism of resistance to nivolumab in KRAS-mutant lung adenocarcinoma. However, the SCLC transformation after targeted therapy followed by immunotherapy has not been reported so far.

The precise mechanism of SCLC transformation in this case was considered from the following aspects. Firstly, it is supposed that there were two histological components of NSCLC and SCLC in the initial tumors before diagnosis, according to the heterogeneity of tumor. Approximately 5%~28% of SCLC contains NSCLC or other pathological components, which was called combined SCLC (9). Some researchers believe that tumor samples from lung biopsy when initial NSCLC diagnosis are insufficient, so the existence of a very low proportion of SCLC components cannot be detected, which is due to the limitations of the existing examination methods and technology (10). As the number of EGFR-mutant NSCLC cells decreased owing to targeted therapy of EGFR-TKI, the SCLC component of initial tumor became dominant. If the initial tumor contained both NSCLC and SCLC components, SCLC components would proliferate and reach PD rapidly after TKI effectively inhibited EGFR-mutant NSCLC components. However, the patient in this case had a relatively long remission and drug response period before SCLC transformation, the hypothesis of mixed histological components may not generalize the whole picture of this case.

Secondly, EGFR-TKI might be the cause of SCLC transformation in this case. The phenomenon of SCLC transformation was firstly described by Zakowski et al, 2006; they reported that a 45-year-old non-smoking female patient with adenocarcinoma received EGFR-TKI without gene analysis, underwent PD after 18 months and received re-biopsy suggesting a synaptophysin-positive SCLC with EGFR 19del (11). Since then, SCLC transformation was gradually recognized and was considered to be one of mechanisms of EGFR-TKI resistance. The transformation of NSCLC to SCLC often occurs 13~18 months after targeted therapy (1, 2, 12). The patients often retain the original EGFR mutation, but the expression of EGFR protein decreases and patients are no longer sensitive to the original TKI treatment (13). The biological behavior of SCLC transformation is similar to that of primary SCLC. Genomic analysis revealed that TP53 and RB1 mutation existed in most SCLC transformation (1, 14). Compared with primary SCLC, patients with SCLC transformation are younger; the proportion of non-smokers or light smokers is higher; the incidences of men and women are similar (1). At present, there is a lack of standard treatment options for SCLC transformation, and chemotherapy of etoposide and platinum for primary SCLC is the mostly used treatment option, which is usually short-term effective. After the transformation of NSCLC to SCLC, the median PFS is around 3.5 months and the median OS is about 10 months (1, 2, 12, 15). There was no significant difference of PFS and OS after SCLC transformation between patients initially receiving the 1st- or 2nd-generation of TKI and the 3rd-generation of TKI (2). In the current case, resistance to 1st-generation TKI occurred 7 months after diagnosis; then the patients received chemotherapy and immunotherapy and SCLC transformation occurred about 7 months later. The transformation period was not consistent with the transformation after TKI resistance mentioned above. The patient in this case acquired EGFR T790M mutation after SCLC transformation, which is different from patients suffering transformation of NSCLC to SCLC after EGFR-TKI treatment retaining the original EGFR mutation.

Additionally, it is hypothesized that NSCLC in this case underwent histological transformation to SCLC owing to immunotherapy of PD-1 inhibitor. Although the mechanism for this is unclear, some theories support this hypothesis. The existence of cancer stem cells, being related to the differentiation and proliferation of cancer cells, were supposed to have ability to differentiate into either SCLC or NSCLC (16–20). Lung adenocarcinoma and SCLC were found to have the potential shared cell of origin. Alveolar type II cells were always believed to be the origin of lung adenocarcinoma. Surprisingly, it was reported that alveolar type II cells could also led to the development of SCLC when targeted disrupting of TP53 and RB1 (21, 22). In this case, it was regrettable that the patient did not undergo next-generation sequencing (NGS) tests. Although the information about TP53 and RB1 mutations at diagnosis was not collected, the possibility that immunotherapy led to alveolar type II cells induced SCLC transformation by changing the tumor microenvironment cannot be excluded.

In summary, we reported a case of a 50-year-old man with EGFR 19del advanced NSCLC, who showed a favorable response to the first-line erlotinib with PR, overcame the resistance to erlotinib with EGFR T790M (–) by a combination of pemetrexed and carboplatin plus PD-1 inhibitor and achieved PR, overcame progression and histologic transformation from NSCLC to SCLC by EC chemotherapy with the main lesion significantly shrinking while metastatic nodules increasing. The pathology of the metastatic nodule showed NSCLC with EGFR T790M (+), which revealed the heterogeneity of tumor. Finally, EC chemotherapy combined with osimertinib was used, and patients responded well. Transformation of NSCLC to SCLC is a result of tumor evolution during the anti-tumor treatment. Although SCLC transformation after immunotherapy is very rare, and its exact molecular mechanism is still under study, it should be paid attention to when disease progression occurred during immunotherapy. Chemotherapy of etoposide and platinum is the common treatment for SCLC transformation, but more effective strategies are need to be explored. What’s more, re-biopsy played a key role in this case, which provided the patient with precise treatment. Therefore, this case suggested that, based on the consideration of tumor evolution and tumor heterogeneity, re-biopsy is of great significance, which could bring more benefits to patients.

## Data availability statement

The original contributions presented in the study are included in the article/supplementary material. Further inquiries can be directed to the corresponding author.

## Ethics statement

The studies involving human participants were reviewed and approved by Medical Ethics Committee of Shanghai Pulmonary Hospital. The patients/participants provided their written informed consent to participate in this study. Written informed consent was obtained from the individual(s) for the publication of any potentially identifiable images or data included in this article.

## Author contributions

M-HY and WL was involved in the drafting and preparation of the manuscript, JY and C-LC were involved in the editing and fact checking of the manuscript. All authors contributed to the article and approved the submitted version.
